# Bibliometric analysis of research on intestinal flora and primary biliary cholangitis published between 2004 and 2024 using VOSviewer and CiteSpace visualization

**DOI:** 10.3389/fmed.2025.1565778

**Published:** 2025-05-21

**Authors:** Tao Li, Wang Jing

**Affiliations:** ^1^Graduate School of Baotou Medical College, Baotou, China; ^2^Department of Gastroenterology, The Second Affiliated Hospital of Baotou Medical College, Baotou, China

**Keywords:** intestinal flora, primary biliary cholangitis, bibliometrics, VOSviewer, CiteSpace

## Abstract

**Background:**

Increasing evidence suggests that the onset and progression of primary biliary cholangitis (PBC) are closely linked to changes in gut microbiota, including bacterial translocation, molecular mimicry, and immune regulation. This study aimed to conduct a bibliometric analysis of the frontiers and hotspots of research on the relationship between gut microbiota and PBC between 2004 and 2024.

**Methods:**

A bibliometric study was conducted by searching the Web of Science database for articles on intestinal flora and PBC published between 2004 and 2024. Excel, VOSviewer, and CiteSpace were used for econometric analysis and visualization of the identified articles.

**Results:**

Between 2004 and 2024, 167 articles focusing on intestinal flora and PBC were published. The number of publications in this field maintained an upward trend over the years, with China and the United States contributing the highest number of articles. The United States had the highest total number of citations, and the institution with the most publications in the United States was the University of California Davis, with the team led by Professor Gershwin contributing the greatest number of articles. *Frontiers in Immunology* had the highest number of articles in the field, while *Nature* had the highest impact in terms of publications in this area of research. The main keywords were “primary sclerosing cholangitis,” “bile acids,” “ursodeoxycholic acid,” “cirrhosis,” “farnesoid X receptor,” “inflammatory bowel disease,” “risk factors,” and “liver disease.”

**Conclusion:**

There is a correlation between gut microbiota and PBC, and the role of gut microbiota in the pathogenesis and treatment of PBC will continue to be a future research direction. Targets such as bile acids and farnesoid X receptors are also current research hotspots.

## Introduction

Primary biliary cholangitis (PBC) is an autoimmune liver disease characterized by progressive intrahepatic bile duct injury and cholestasis (1). PBC was formerly known as primary biliary cirrhosis but was renamed as *primary biliary cholangitis* by the liver research community between 2014 and 2015 to more accurately reflect the characteristics of the disease (2). PBC has a high prevalence in middle-aged women, with a reported female-to-male ratio ranging from 4–5:1 to 9–10:1 (3). Anti-mitochondrial antibody (AMA) is a specific serum marker for PBC and serves as an important diagnostic indicator. Approximately 90%−95% of patients with PBC are diagnosed based on a positive AMA result. However, AMA serum levels are not directly associated with disease severity (4). Specific antinuclear antibodies, such as Sp100 and gp210, as well as anti-kelch-like-12 and antihexokinase-1 antibodies, are useful for diagnosing PBC in AMA-negative patients (5). Clinically, PBC primarily manifests with symptoms such as fatigue and itching or jaundice, with progression to liver fibrosis as the disease develops, eventually leading to cirrhosis and liver failure (6). The pathogenesis of PBC is complex and involves the interaction of environmental, genetic, and immunologic factors (7, 8). Ursodeoxycholic acid (UCDA) is currently recognized as the first-line treatment for PBC; however, there is no consensus on the optimal treatment for PBC in cases with a poor response to UCDA. Obeticholic acid (OCA), fibrates, and budesonide have been adopted as second-line treatment options in some countries (6, 9, 10). With advances in the understanding of PBC, recent studies have shown that the intestinal flora plays a key role in its development and progression through mechanisms such as bacterial translocation, molecular mimicry, and immune regulation, highlighting the potential of the intestinal flora as a diagnostic marker and novel therapeutic target for PBC (11–13).

Bibliometrics is a discipline that integrates mathematics and statistics to evaluate research and future trends within a specific field. It identifies research hotspots and predicts future development directions across different time periods using qualitative and quantitative analyses of different characteristics of publications in the field of research including countries, institutions, journals, authors, and keywords; notably, bibliometric analysis offers advantages that are distinct from the traditional methods of review, meta-analysis, or experimental research (14–16). Here, we conducted a bibliometric analysis of studies on intestinal flora and PBC published between 2004 and 2024 to identify research hotspots and trends in this field.

## Materials and methods

### Data sources and search strategy

**Figure 1:**
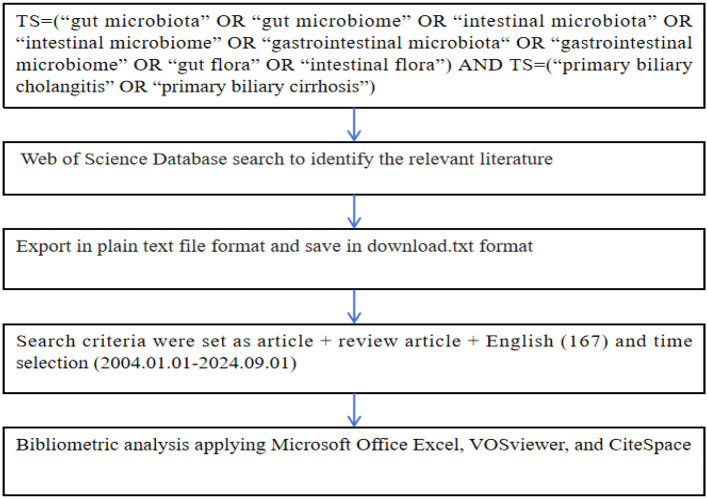
Flow chart of the literature search strategy.

On August 11, 2024, we utilized the Web of Science database to perform an advanced search of the literature on intestinal flora in PBC covering the period from January 1, 2004, to September 1, 2024. The following subject terms were entered: TS = (“gut microbiota” OR “gut microbiome” OR “intestinal microbiota” OR “intestinal microbiome” OR “gastrointestinal microbiota” OR “gastrointestinal microbiome” OR “gut flora” OR “intestinal flora”) AND TS = (“primary biliary cholangitis” OR “primary biliary cirrhosis”). A total of 170 relevant articles were identified. The inclusion criteria were as follows: (1) Article type: articles or review articles; and (2) Literature published in English. One-hundred and sixty-seven articles that met the above requirements were exported in plain text file format; the recorded content included the full records and cited references. Finally, the files were saved in download.exe format. The specific search process is detailed in Figure 1.

### Data analysis and visualization

This study used CiteSpace software, developed by Dr. Chaomei Chen and a team at DeSales University (United States), and VOSviewer software, developed at Leiden University (The Netherlands), for the bibliometric analysis.

VOSviewer is a software tool for creating and exploring maps based on network data. It connects co-authorship, co-occurrences, citations, bibliographic coupling, and co-citation links in the form of a network, overlay, or density visualization and builds a visual map of the literature dynamics and connecting paths. Vosviewer is a useful tool for researchers to understand the structure and dynamics of a research field and is currently one of the most popular and promising visualization tools for bibliometric analysis (17, 18).

CiteSpace is a visual bibliometrics tool that can efficiently visually describe the structure, distribution, and trends in the literature in the form of visual maps (19). CiteSpace was applied in this study to analyze the journal dual map overlay, co-cited references, and keywords.

## Results

### Overview of the number of publications

**Figure 2:**
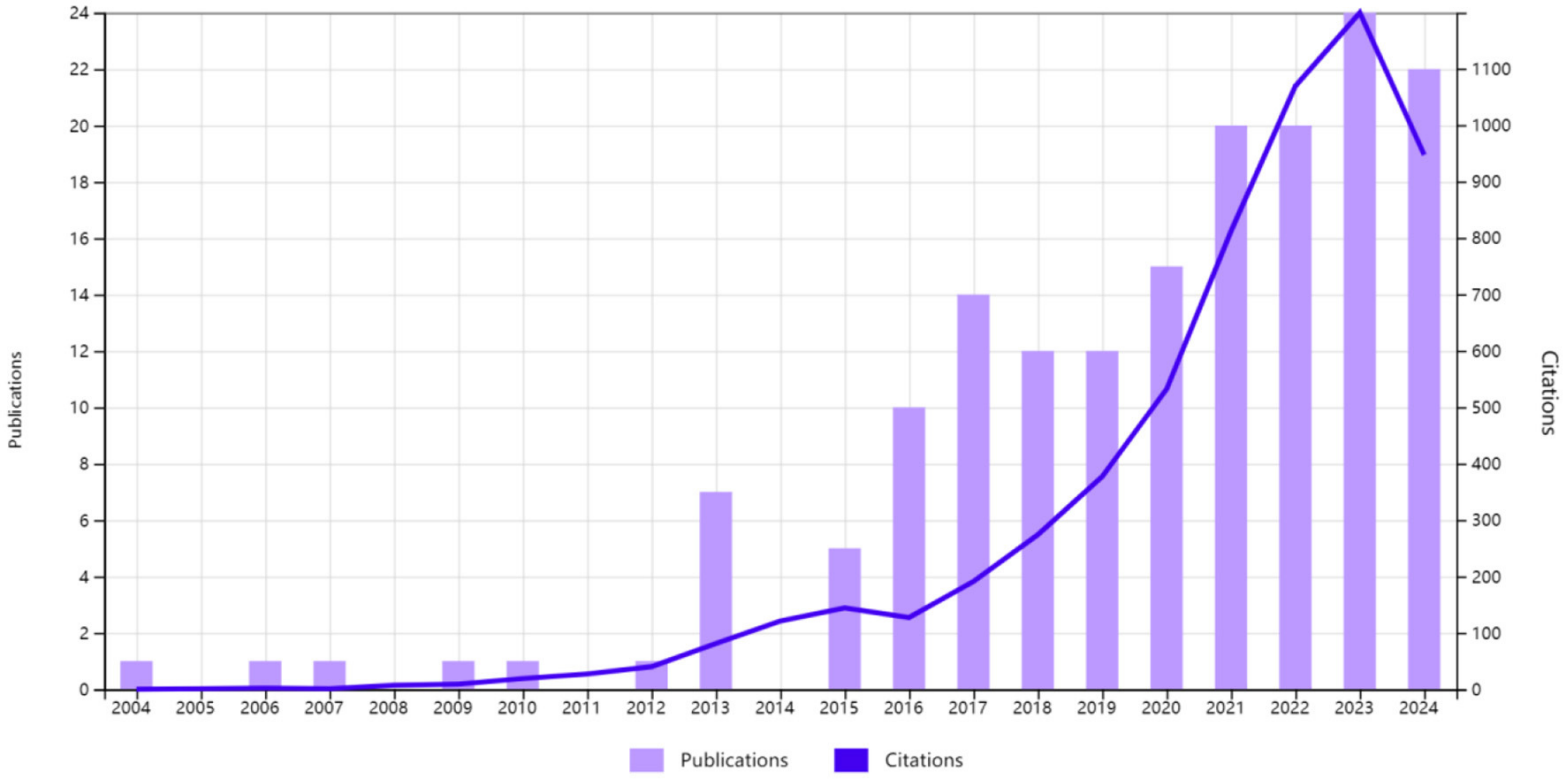
Trends in publications and the number of citations related to intestinal flora and primary biliary cholangitis.

We identified 167 publications on intestinal flora and PBC published between 2004 and 2024. Of these, 68 were experimental articles and 99 were reviews. The number of citations in these articles was 5,984, with an average of 35.83 citations per article. The highest number of citations (*n* = 1,199) was recorded in 2023. The number of relevant publications steadily increased since 2016, which may be related to the continuous development of metabolomics and macro-genome sequencing technology. The largest number of related research articles was published in 2023, with a total of 24 articles. This trend is likely to continue growing, which also indicates that researchers' interest in the study of intestinal flora and its relationship with PBC remains strong (Figure 2).

### Analysis of country/region productivity

**Figure 3:**
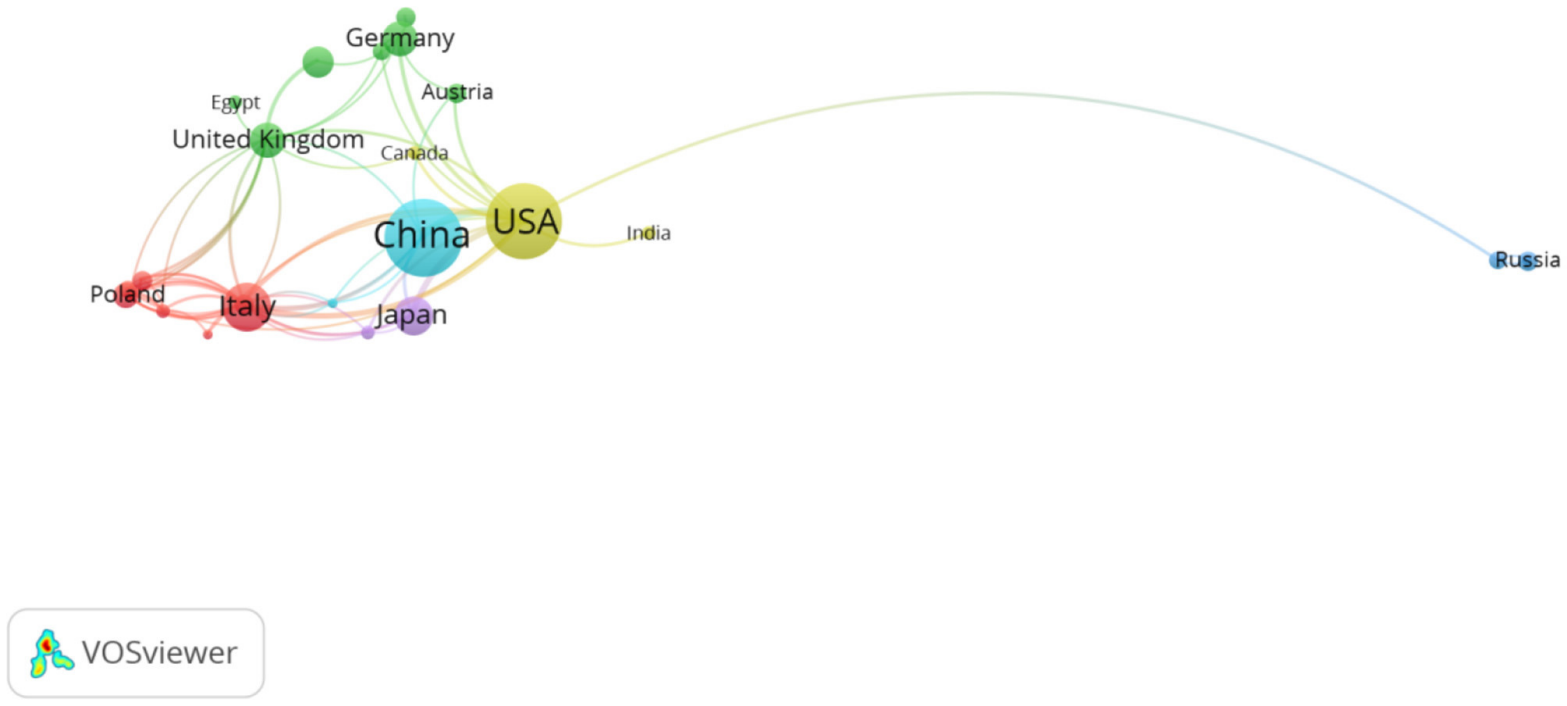
Inter-country cooperation network map.

Before the analysis, we categorized Taiwan as part of China, and Wales and England as parts of the United Kingdom. Figure 3 presents the inter-country collaboration network map created using VOSviewer showing that 28 countries were involved in the research on intestinal flora and PBC. Among the top five countries or regions in terms of the number of publications (Table 1), the leading country or region in research output was China (56 articles), followed by the United States (55 articles), Italy (23 articles), and Japan (13 articles). The United Kingdom and Germany were tied for fifth place (12 articles). The number of articles published by China and the United States was far greater than those published by other countries, highlighting the scientific research strength of these two countries in this field. Although China topped the list in terms of the number of articles published, the quality of the articles requires improvement (average citation count: 28.95 times), and the total link strength was not high (20). Therefore, there remains scope for improvement in terms of strengthening cooperation with other countries. The United States had the highest total number of citations (*n* = 3,467) and total link strength (47), emphasizing the country's strong influence and its close cooperation with other countries. Austria had the highest average number of citations (*n* = 82.75) despite its small number of publications (*n* = 4), indicating that the country's scientific strength in the field is widely recognized.

**Table 1:** Top five countries by number of publications.

**Rank**	**Country**	**Number of publications**	**Total citations**	**Average number of citations**	**Total connection strength**
1	China	56	1,621	28.95	20
2	China	55	3,467	63.04	47
3	Italy	23	570	24.78	24.78
4	Japan	15	670	44.67	13
5	Germany	12	607	50.58	8
6	United Kingdom	12	237	19.75	20

### Institutional analysis

**Figure 4:**
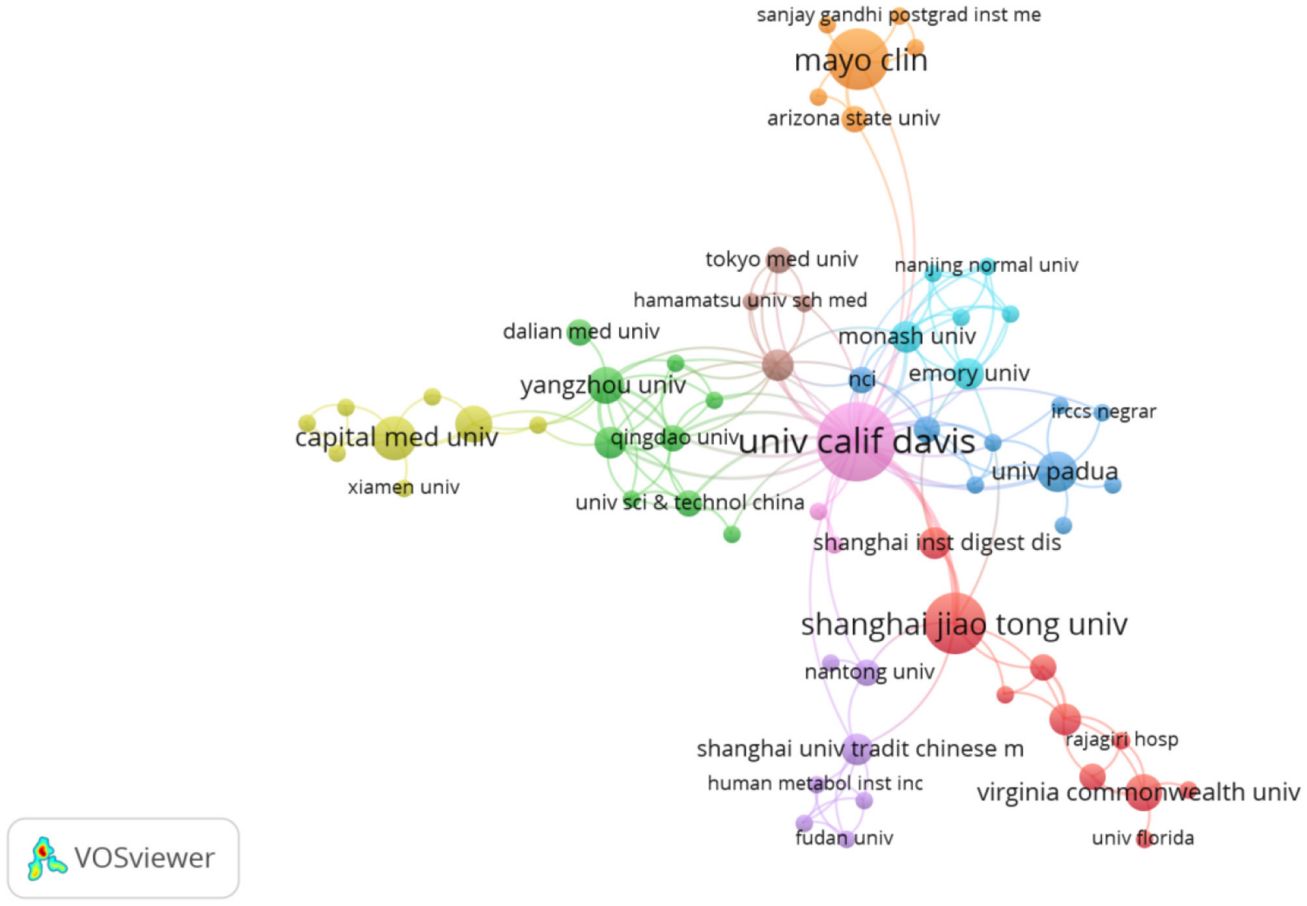
Network diagram of inter-institutional cooperation.

Table 2 shows the top 10 research institutions by number of publications in the field of research on intestinal flora and PBC. The institution with the largest number of publications was the University of California, Davis (United States) (*n* = 18); followed closely by the Mayo Clinic (United States) and Shanghai Jiao Tong University (China), each with 11; the University of Oslo (Norway) (*n* = 9); the University of Birmingham (United Kingdom) (*n* = 7); Oslo University Hospital (Norway) and Capital Medical University (China), each with 6; and the National Hospital (Norway), the University of Padua (Italy), and the University Medical Center Hamburg-Eppendorf (Germany), each with 5. North Carolina State University and Ohio State University (United States) were the two institutions with the highest average number of citations (*n* = 305), followed by the Albert Einstein College of Medicine (United States), Chan Zuckerberg Biohub Network (United States), Daiichi Sankyo Company, Limited (Japan), and Stanford University (United States), each with an average of 275 citations. As shown by the inter institutional cooperation network diagram generated by VOSviewer (Figure 4), research institutions in developed countries, led by the United States, work in close cooperation. Further, although there is cooperation between China and these research institutions, there is still considerable room for improvement at this level. Close cooperation between institutions can better promote the development of this field.

**Table 2:** Top 10 institutions with the largest number of publications.

**Rank**	**Name of institution**	**Number of publications**	**Total number of citations**	**Average number of citations**	**Country**	**Total link strength**
1	University of California, Davis	18	887	49.28	USA	52
2	Mayo Clinic	11	484	44	USA	7
3	Shanghai Jiao Tong University	11	772	70.18	China	15
4	University of Oslo	9	225	225	Norway	19
5	University of Birmingham	7	82	11.71	United Kingdom	23
6	Capital Medical University	6	31	5.17	China	8
7	Oslo University Hospital	6	147	24.5	Norway	14
8	National Hospital Norway	5	138	27.6	Norway	11
9	University Medical Center Hamburg-Eppendorf	5	239.5	47.8	Germany	4
10	University of Padua	5	200	40	Italy	10

### Author analysis

**Figure 5:**
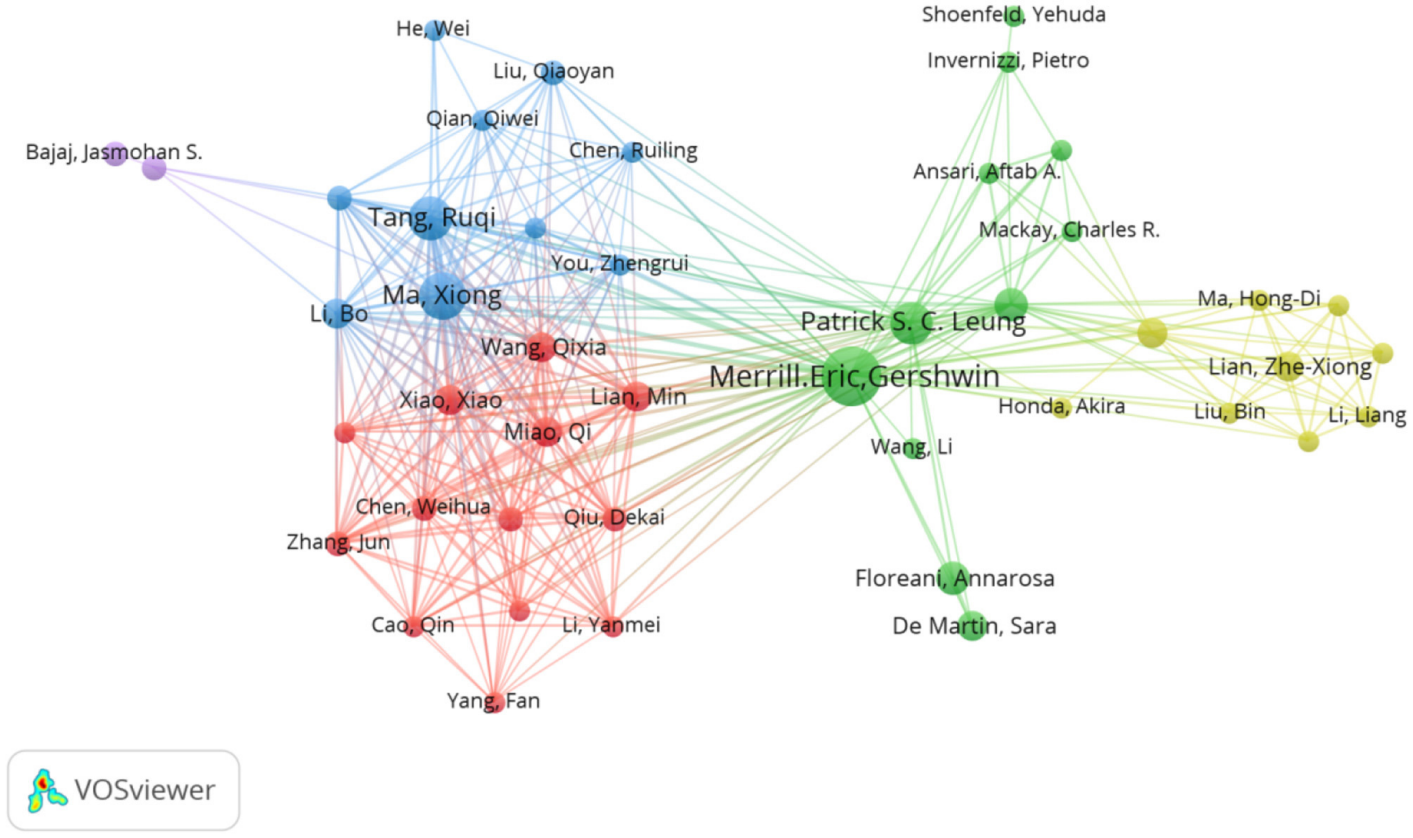
Network diagram of the cooperation between authors.

Using VOSviewer to analyze the authors, Figure 5 shows the collaborative network graph of authors in these research areas. The size of the circle in the graph represents the number of articles published by the authors, the clusters between nodes of the same color represent the collaborative relationship between authors, and the connecting lines between clusters represent the collaborative relationship between teams. A total of 833 authors published relevant articles in this field, with 21 authors having published more than 4 articles. Table 3 lists the top five authors with the highest number of publications. Merrill Eric Gershwin had the highest number of publications in the field (*n* = 16), followed by Ma Xiong (*n* = 10), Tang Ruqi (*n* = 9), Patrick S. C. Leung (*n* = 8), and Albert J. Czaja (*n* = 6). These authors were found to have strong collaborative relationships with each other, suggesting that close collaboration is conducive to the advancement of scientific research in this field. The number of citations represents the recognition of the article. The team with the highest total number of citations was that led by Professor Merrill Eric Gershwin (*n* = 874), followed by that led by Professor Ma Xiong (*n* = 758) and that led by Professor Tang Ruqi (*n* = 756). These teams also had the highest number of publications, reflecting their influence in the field. Of note, the articles with the highest average citation rates were those co-authored by Cao Qin, Fan Zhuping, and Li Yanmei (180.5 citations), followed by Yang Fan (152 citations), and Shi Ding (139.5 citations). This indicates that the scientific contributions of these authors have garnered attention and recognition from their peers.

**Table 3:** Top five authors with the highest number of publications in the field.

**Rank**	**Authors**	**Number of articles**	**Total number of citations**	**Average number of citations per article**
1	Merrill Eric Gershwin	16	874	54.625
2	Ma Xiong	10	758	75.8
3	Tang Ruqi	9	756	84
4	Patrick S. C. Leung	8	373	46.625
5	Albert J. Czaja	6	146	24.333

### Analysis of publications

**Figure 6:**
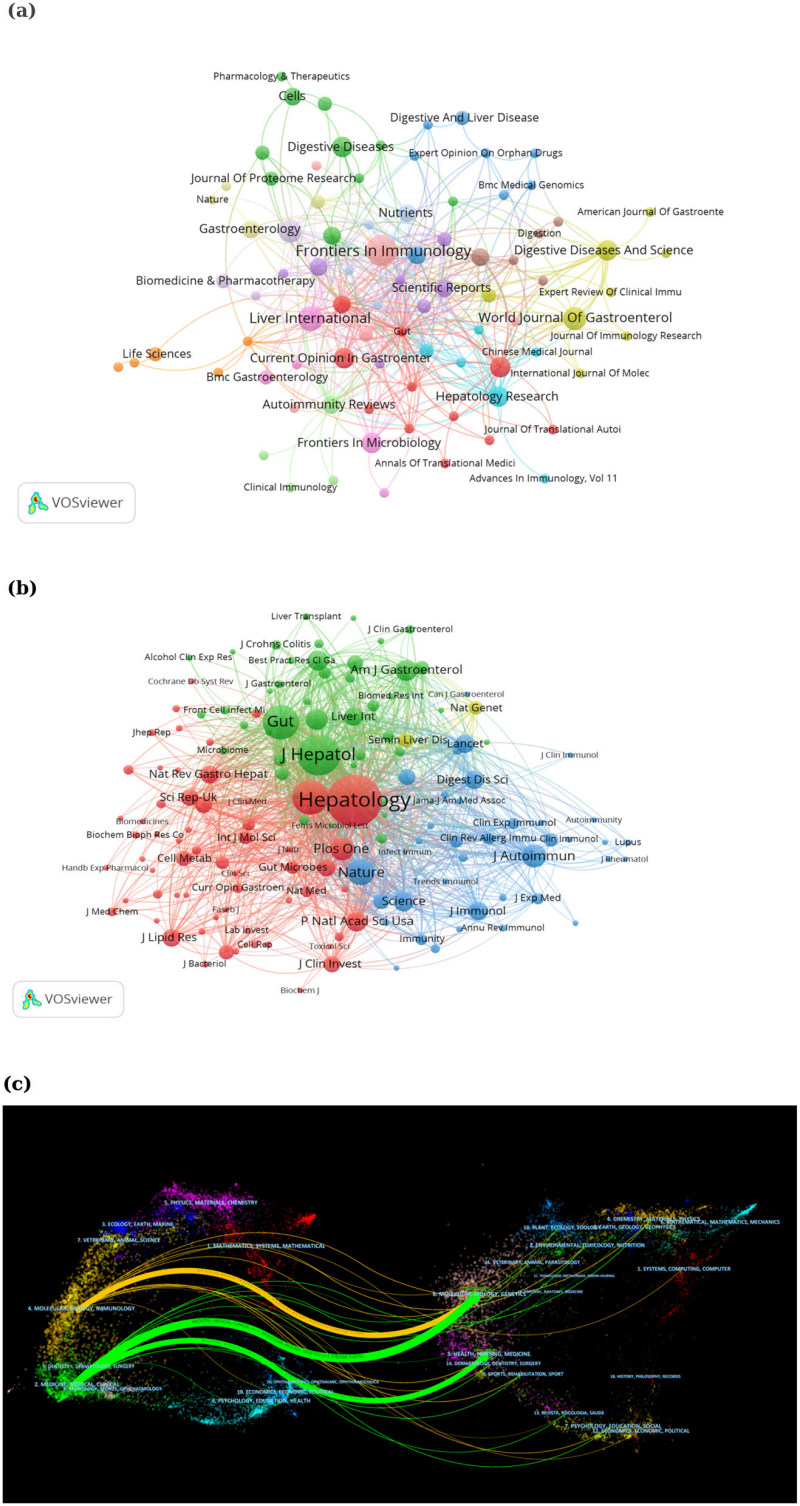
**(a)** Journal overlay of studies on intestinal flora and PBC. **(b)** Co-cited journal network co-occurrence map. **(c)** Co-occurrence map of journal networks. PBC, primary biliary cholangitis.

Ninety-five journals published articles on intestinal flora and PBC (Figure 6a). Table 4 lists the top 10 journals in terms of productivity. *Frontiers in Immunology* (*n* = 9, impact factor [IF]: 5.7; Q1) emerged as the most productive journal, followed by *Liver International* (*n* = 6, IF: 6, Q1), the *World Journal of Gastroenterology* (*n* = 5, IF: 4.3, Q1), and the *International Journal of Molecular Sciences* (*n* = 5, IF: 4.9, Q1). The most frequently cited journal was the *Journal of Lipid Research* (*n* = 359, IF: 5, Q1), followed by *Nature* (*n* = 275, IF: 50.5, Q1) and *Gut* (*n* = 269, IF: 23.1, Q1). In the analysis of co-cited journals (Figure 6b), 143 journals with more than 20 co-citations were identified. The top 10 most co-cited journals are listed in Table 5; *Hepatology* was the most co-cited (IF: 13, Q1), followed by the *Journal of Hepatology* (IF: 26.8, Q1), *Gut* (IF: 23.1, Q1), *Gastroenterolog*y (IF: 26.3, Q1), and *Nature* (IF: 50.5, Q1). The journal with the highest impact was *Nature* (IF: 50.5, Q1), followed by *Nature Reviews Gastroenterology & Hepatology* (IF: 46.4, Q1), and *Gastroenterology* (IF: 26.3, Q1). The journal double chart overlay is a useful tool to present the citation relationship between disciplines, thus helping researchers to better understand the frontiers and trends in related research areas (20). The left side of the chart shows the citation relationship between the cited literature and the cited journal. Moreover, it shows the journals in which the citing literature is located, representing the main disciplines included in the research field. The right side of the chart shows the journals in which the cited literature is located, representing the relevant disciplines cited. The thickened orange and green lines represent the main pathways connecting the citing journals to the cited journals. Figure 6c shows that the literature cited in MOLECULAR/BIOLOGY/IMMUNOLOGY journals was primarily published in MOLECULAR/BIOLOGY/GENETICS journals, whereas research articles from MEDICINE/MEDICAL/CLINICAL journals frequently cited literature from MOLECULAR/BIOLOGY/GENETICS as well as from HEALTH/NURSING and MEDICAL journals.

**Table 4:** Top 10 most productive journals.

**Rank**	**Journal**	**Number of articles**	**Total number of citations**	**Average number of citations**	**Impact factor**	**Journal citation reports**
1	*Frontiers in Immunology*	9	165	18.33	5.7	Q1
2	*Liver International*	6	185	30.83	6.0	Q1
3	*World Journal of Gastroenterology*	5	119	23.8	4.3	Q1
4	*International Journal of Molecular Sciences*	5	91.0	18.2	4.9	Q1
5	*Hepatology Research*	4	163	40.75	3.9	Q1
6	*Journal of Autoimmunity*	4	276	69	7.9	Q1
7	*Digestive Diseases and Sciences*	4	116	29	2.5	Q3
8	*Frontiers in Microbiology*	4	17	4.25	4.0	Q2
9	*Current Opinion in Gastroenterology*	4	8.75%	8.75	2.6	Q2
10	*Digestive Diseases*	4	39	9.75	2.0	Q3

**Table 5:** Top 10 most co-cited journals.

**Rank**	**Journal**	**Total number of citations**	**Impact factor**	**Journal citation reports**
1	*Hepatology*	1,693	13	Q1
2	*Journal of Hepatology*	1,043	26.8	Q1
3	*Gut*	754	23.1	Q1
4	*Gastroenterology*	746	26.3	Q1
5	*Nature*	450	50.5	Q1
6	*Journal of Autoimmunity*	339	7.9	Q1
7	*Plos One*	321	2.9	Q1
8	*American Journal of Gastroenterology*	284	8.5	Q1
9	*World Journal of Gastroenterology*	280	4.3	Q1
10	*Alimentary Pharmacology & Therapeutics*	264	6.6	Q1

### Literature citation and co-citation analysis

**Figure 7:**
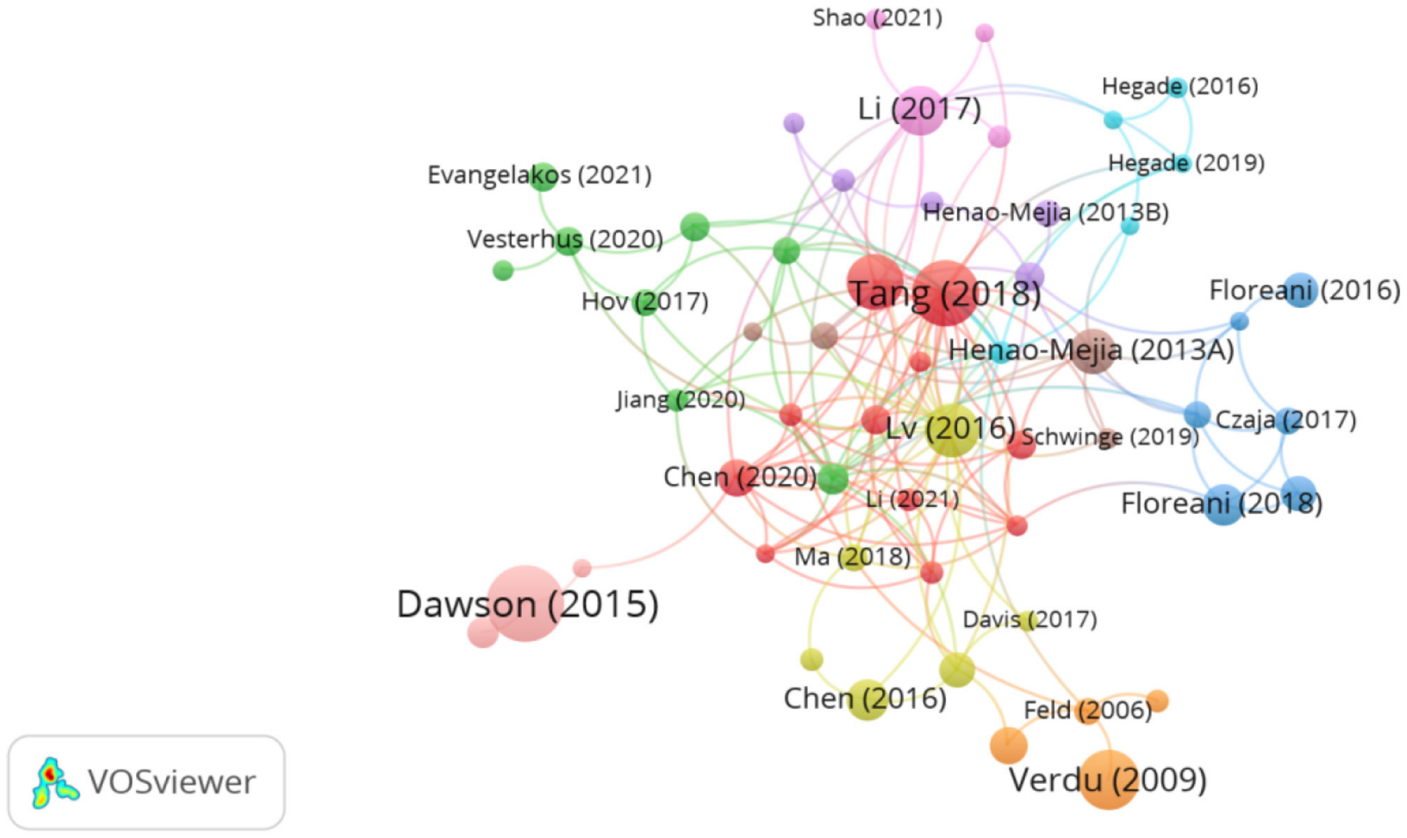
Network visualization of the highly cited literature analysis.

Figure 7 depicts a network diagram of the 75 publications with 20 citations or more, including 6 reviews, 2 basic studies, and 2 clinical trials in the top 10 cited publications (Table 6). The most cited article was entitled “Intestinal transport and metabolism of bile acids” (*n* = 359) by Dawson et al. This article focuses on the intestinal transport and metabolism of bile acids and the impact of the intestinal flora on bile acid signaling (21). “Diversification of host bile acids by members of the gut microbiota” by Winston et al. was the second most cited article (*n* = 305) (22). The study covers four main areas: (1) the ability of gut flora to diversify host bile acids; (2) assessment of how gut microbes affect the bile acid pool and, in turn, regulate the structure of the gut microbial community; (3) comparison of differences in the distribution of bile acids between species; and (4) the effect of UCDA on the host bile acid pool through the microbial-bile acid-host axis and its impact on gut microbiota. The article highlights the influence of gut microbes on bile acids and proposes that restoring host and gastrointestinal health can be achieved by regulating the gut microbe-bile acid-host axis. Funabashi et al. (23) published “A metabolic pathway for bile acid dehydroxylation by the gut microbiome,” which has been cited 275 times. In this study, the author reconstructed the bile acid 7 α-dehydroxylation pathway *in vitro*, confirming that microorganisms can regulate the metabolic pathways of bile acids. Together, these findings provide strong evidence for targeting the intestinal flora for the treatment of PBC. These highly cited literature are mainly distributed in Q1, reflecting the influence of these publications.

**Table 6:** Top 10 articles with the highest number of citations.

**Rank**	**Title**	**Journal**	**First author**	**Number of citations**	**Type of literature**	**Year of publication**	**Journal citation reports**
1	Intestinal transport and metabolism of bile acids	*Journal of Lipid Research*	Paul A. Dawson	359	Review	2015	Q1
2	Diversification of host bile acids by members of the gut microbiota	*Gut Microbes*	Jenessa A. Winston	305	Review	2020	Q1
3	A metabolic pathway for bile acid dehydroxylation by the gut microbiome	*Nature*	Masanori Funabashi	275	Basic research	2020	Q1
4	Gut microbial profile is altered in primary biliary cholangitis and partially restored after UDCA therapy	*Gut*	Tang Ruqi	269	Clinical trial	2018	Q1
5	Between celiac disease and irritable bowel syndrome: the “no man's land” of gluten sensitivity	*American Journal of Gastroenterology*	Elena F. Verdu	224	Review	2009	Q1
6	Gut microbiome, liver immunology, and liver diseases	*Cellular & Molecular Immunology*	Wang Rui	194	Review	2021	Q1
7	Gut microbiome, liver immunology, and liver diseases	*Nature Reviews Gastroenterology & Hepatology*	Lulu Sun	194	Review	2021	Q1
8	Alterations and correlations of the gut microbiome, metabolism and immunity in patients with primary biliary cirrhosis	*Environmental Microbiology*	Lv Long-Xian	175	Clinical trial	2016	Q2
9	Intestinal microbes affect phenotypes and functions of invariant natural killer T cells in mice	*Gastroenterology*	Gerhard Wingender	166	Basic research	2012	Q1
10	Bile acids and intestinal microbiota in autoimmune cholestatic liver diseases	*Autoimmunity Reviews*	You Li	153	Review	2017	Q1

**Figure 8:**
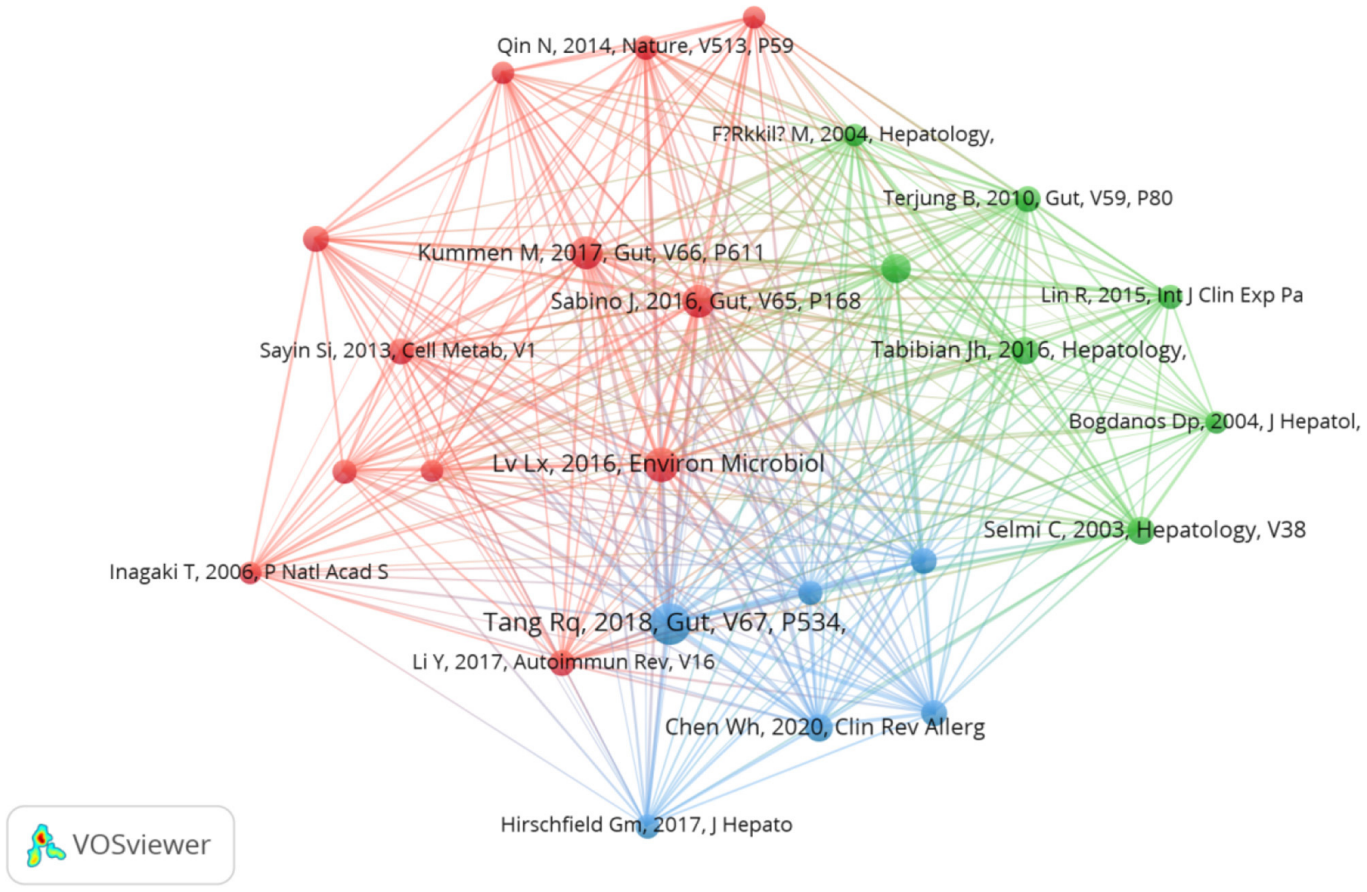
Visual network diagram of the co-cited documents (cited ≥20 times).

Co-cited literature refers to two or more articles that are cited by another article simultaneously (24). The analysis of co-cited literature can provide a quick understanding of the current research frontiers in the field (17). We retrieved 12,421 co-cited literature from the past 20 years on the role of gut microbiota in PBC. VOSviewer software analysis identified 25 references with 20 or more co-citations (Figure 8). The top 10 most co-cited references are listed in Table 7. The article published in 2018 by Tang et al. (25) in *Gut Microbiota* titled “Gut microbial profile is altered in primary biliary cholangitis and partially restored after UDCA therapy” was the most co-cited article (64 co-citations). In the cross-sectional and prospective study, the authors conducted a systematic comparative analysis of the gut microbiome of patients with PBC and healthy controls. The results revealed that patients with PBC develop dysbiosis of the intestinal flora, which is partially alleviated by ursodeoxycholic acid (UDCA) treatment, suggesting that the intestinal microbiota may serve as a potential novel diagnostic biomarker and therapeutic target for PBC. The second most cited article, titled “Alterations and correlations of the gut microbiome, metabolism and immunity in patients with primary biliary cirrhosis,” was published in 2016 in *Environmental Microbiology* by Tang et al. (25). The authors compared and analyzed the serum, urine, and feces of patients with PBC and of healthy individuals. Compared to that of healthy individuals, the gut microbiota of patients with PBC exhibited specific changes related to inflammatory factors, liver injury indicators, and metabolic abnormalities. Moreover, the relationships between certain microorganisms and immunity and metabolism had also undergone changes, indicating that the gut microbiota play an important role in the occurrence or development of PBC. Lv et al. (12) conducted a study on the association of primary sclerosing cholangitis (PSC) with intestinal flora and inflammatory bowel disease (IBD). In their article, titled “Primary sclerosing cholangitis is characterized by intestinal dysbiosis independent from IBD,” published in *Gut* in 2016, the authors suggested that alterations in intestinal flora, which occur independently of IBD, play an important role in the pathogenesis of PSC and that the modulation of intestinal flora can be used to treat or prevent PSC.

**Table 7:** Top 10 most co-cited articles.

**Rank**	**Title**	**Journal**	**First author**	**Total number of citations**	**Year of publication**
1	Gut microbial profile is altered in primary biliary cholangitis and partially restored after UDCA therapy	*Gut Microbiota*	Tang Ruqi	64	2018
2	Alterations and correlations of the gut microbiome, metabolism and immunity in patients with primary biliary cirrhosis	*Environmental Microbiology*	Lv Long-Xian	46	2016
3	Primary sclerosing cholangitis is characterized by intestinal dysbiosis independent from IBD	*Gut*	João Sabino	43	2016
4	The gut microbial profile in patients with primary sclerosing cholangitis is distinct from patients with ulcerative colitis without biliary disease and healthy controls	*Gut*	Martin Kummen	40	2017
5	Randomized clinical trial: vancomycin or metronidazole in patients with primary sclerosing cholangitis - a pilot study	*Aliment Pharmacology & Therapeutics*	J. H. Tabibian	32	2013
6	Comprehensive Analysis of Serum and Fecal Bile Acid Profiles and Interaction with Gut Microbiota in Primary Biliary Cholangitis.	*Clinical Reviews in Allergy & Immunology*	Weihua Chen	31	2020
7	Patients with primary biliary cirrhosis react against a ubiquitous xenobiotic-metabolizing bacterium	*Hepatology*	Carlo Selmi	28	2003
8	Absence of the intestinal microbiota exacerbates hepatobiliary disease in a murine model of primary sclerosing cholangitis	*Hepatology*	James H. Tabibian	28	2016
9	Gut microbiota regulates bile acid metabolism by reducing the levels of tauro-beta-muricholic acid, a naturally occurring FXR antagonist	*Cell Metabolism*	Sama I. Sayin	27	2013
10	Gut dysbiosis associated with clinical prognosis of patients with primary biliary cholangitis	*Hepatol Research*	Masanori Furukawa	26	2020

**Figure 9:**
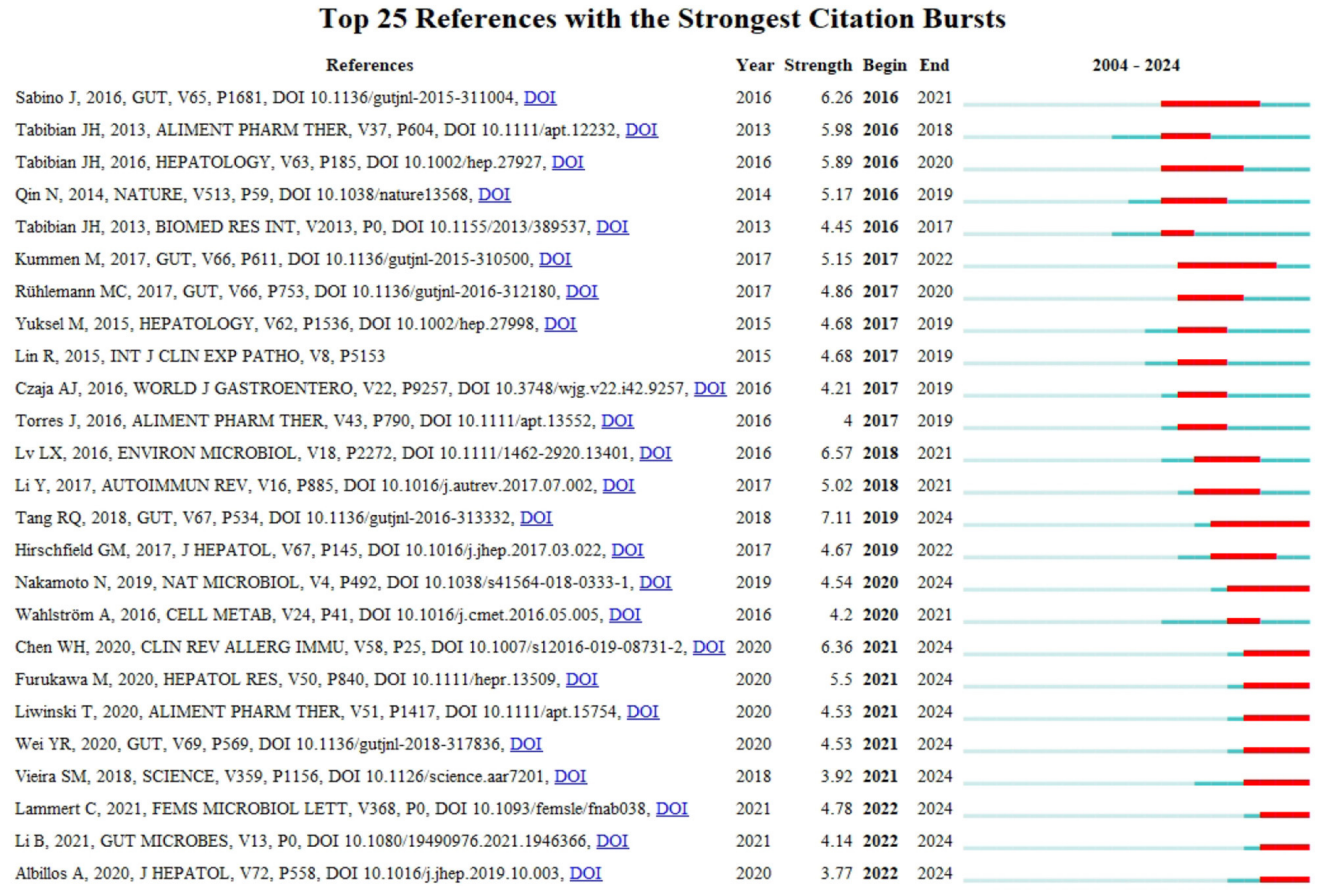
Top 25 articles with the highest outbreak intensity.

Burst detection of cited references represents the direction of research in a particular field (27). Figure 9 shows the top 25 references with the strongest citation bursts in the field of intestinal flora and PBC research over the past two decades, as determined using CiteSpace. These references represent the key focus areas and trends in the field. The article titled “Gut microbial profile is altered in primary biliary cholangitis and partially restored after UDCA therapy” ranked first, with the highest outbreak intensity (strength: 7.11). We identified 10 publications that maintained a high citation rate over the past 5 years, indicating sustained research interest in their content.

**Figure 10:**
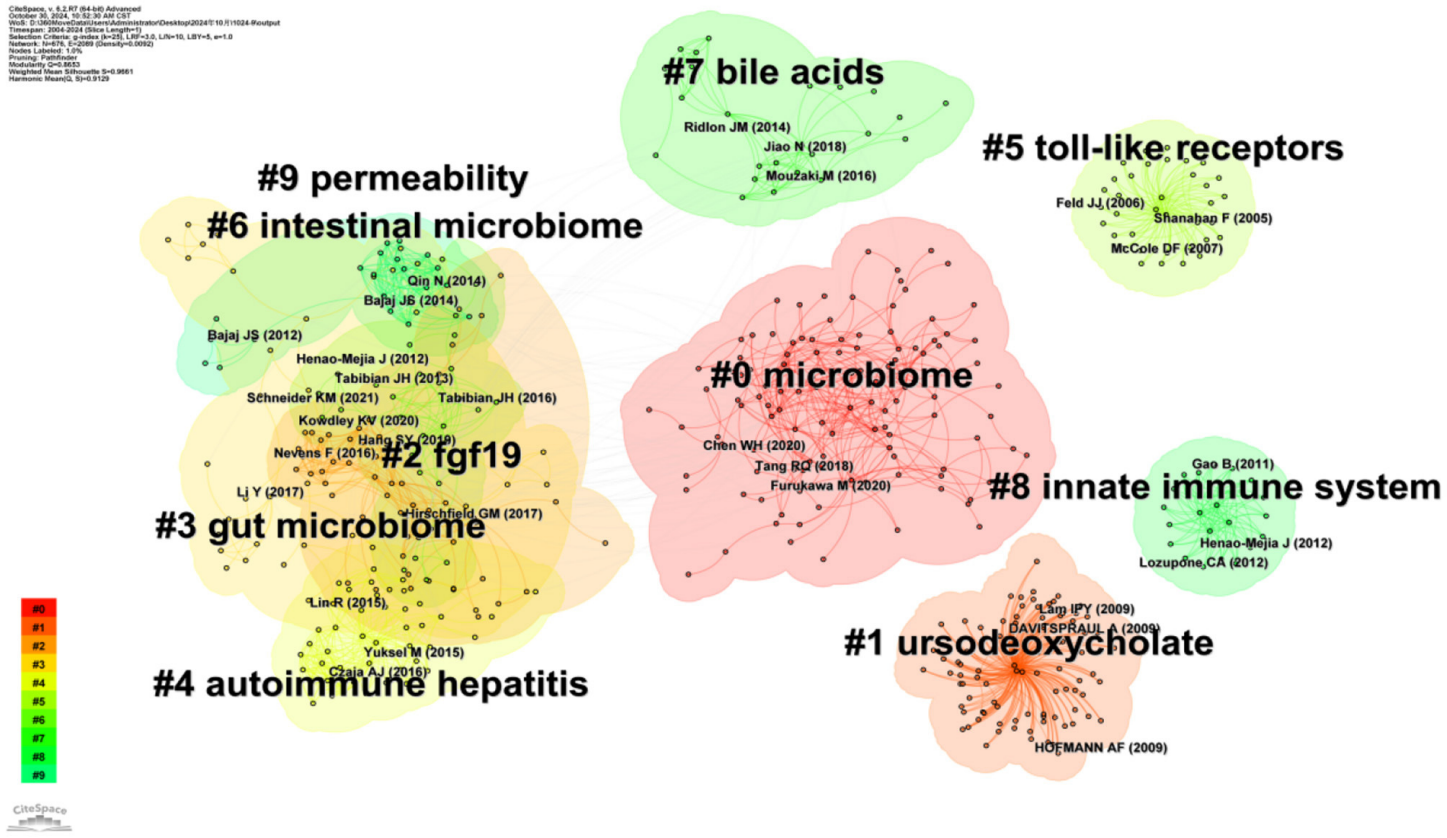
Visual network diagram of the cluster analysis of co-cited documents.

**Figure 11:**
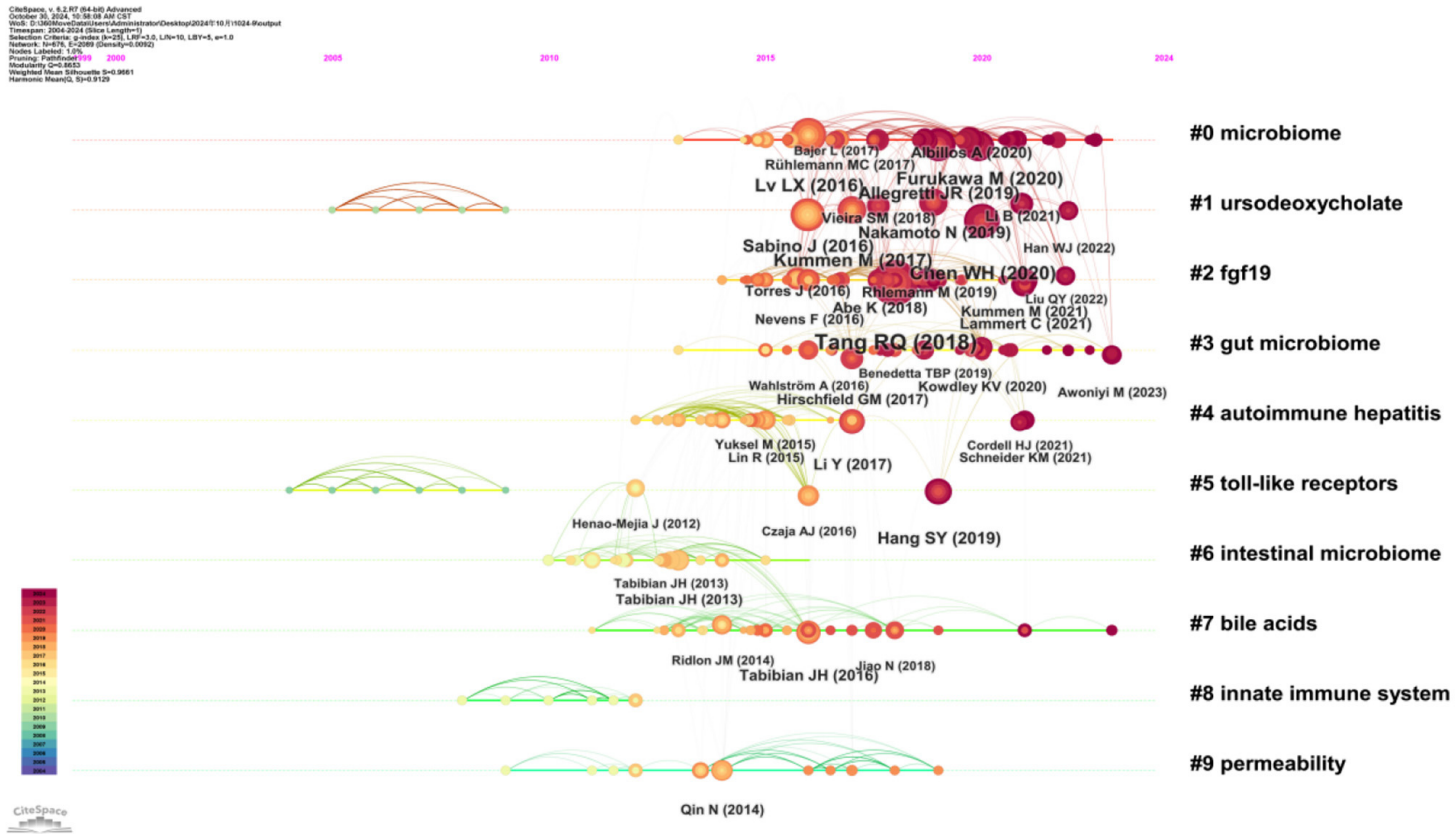
Visual network diagram of the time axis of co-cited reference clustering.

CiteSpace software was used to continue the cluster analysis of the co-cited literature. Figure 10 shows the categorization of the literature into the following ten major clusters (Q = 0.6986, S = 0.834): #0 microbiome, #1 ursodeoxycholate, #2 fgf19, #3 gut microbiome, #4 autoimmune hepatitis, #5 toll-like receptors, #6 intestinal microbiome, #7 bile acids, #8 innate immune system, and #9 permeability. According to the time trend axis (Figure 11), clusters remaining in an active state were: #0 microbiome, #1 ursodeoxycholate, #2 fgf19, #3 gut microbiome, #4 autoimmune hepatitis, #5 toll-like receptors, #6 intestinal microbiome, #7 bile acids, #8 innate immune system, and #9 permeability According to the time trend axis (Figure 11), the clusters that remained active were #0 microbiome, #3 gut microbiome, and #7 bile acids, suggesting that intestinal flora and bile acids will remain research hotspots for the foreseeable future.

### Keyword analysis and research hotspots

**Figure 12:**
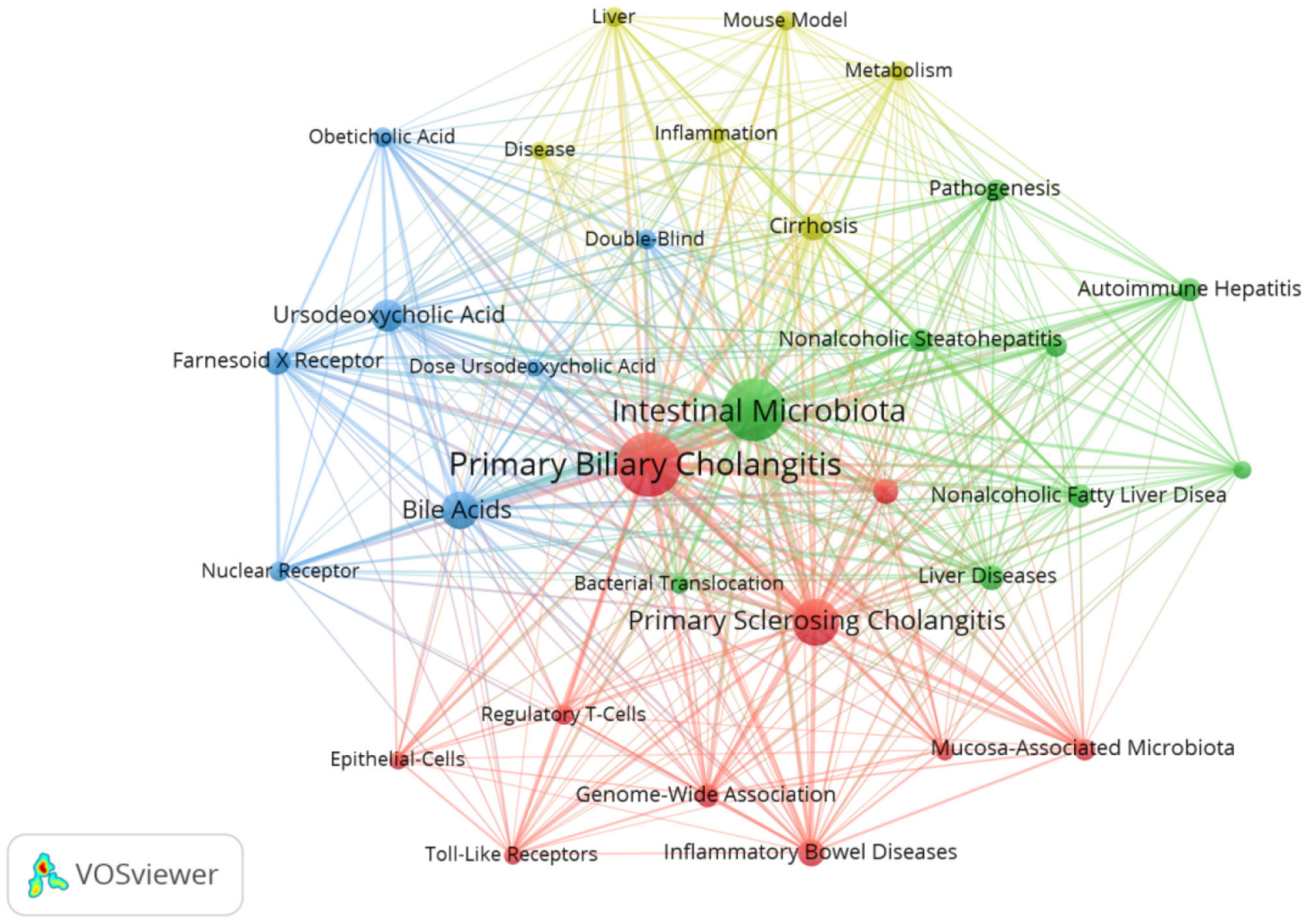
Keyword clustering network graph visualization.

Keywords are the core words used to summarize the content of the literature, and their frequency of occurrence provides insights into research hotspots and future trends within a specific field over recent years (28). Over the past 20 years, 880 high-frequency keywords appeared in the literature, with a minimum of 10 repetitions as the threshold. After synonym adjustment and merging, 32 high frequency keywords were obtained, excluding the two themes of “primary biliary cholangitis” (134) and “intestinal microbiota” (123). The remaining keywords with the highest frequency were “primary sclerosing cholangitis” (68), “bile acids” (45), “ursodeoxycholic acid” (32), “cirrhosis” (25), “farnesoid X receptor” (23), “inflammatory bowel disease” (23), “risk factors” (21), and “liver disease” (20). These keywords were inputted into the VOSviewer software to perform a co-occurrence analysis and generate the corresponding keyword co-occurrence clustering network diagram. Figure 12 shows that these keywords were divided into four clusters. Cluster 1 (red) included “autoimmune liver diseases,” “epithelial cells,” “genome-wide association,” “inflammatory bowel disease,” “mucosa-associated microbiota,” “primary biliary cholangitis,” “primary sclerosing cholangitis,” “regulatory T-cells,” “toll-like receptors,” and “risk factors.” Cluster 2 (green) included “autoimmune hepatitis,” “bacterial translocation,” “dysbiosis,” “hepatocellular carcinoma,” “intestinal microbiota,” “liver disease,” “nonalcoholic fatty liver disease,” “nonalcoholic steatohepatitis,” and “pathogenesis.” Cluster 3 (blue) included “bile acids,” “dose ursodeoxycholic acid,” “double-blind,” “farnesoid X receptor,” “nuclear receptor,” “obeticholic acid,” and “ursodeoxycholic acid.” Cluster 4 (yellow) included “cirrhosis,” “disease,” “inflammation,” “liver,” “metabolism,” and “mouse model.” The clustering analysis indicated a correlation between the gut microbiota and various liver diseases, including primary biliary cholangitis.

**Figure 13:**
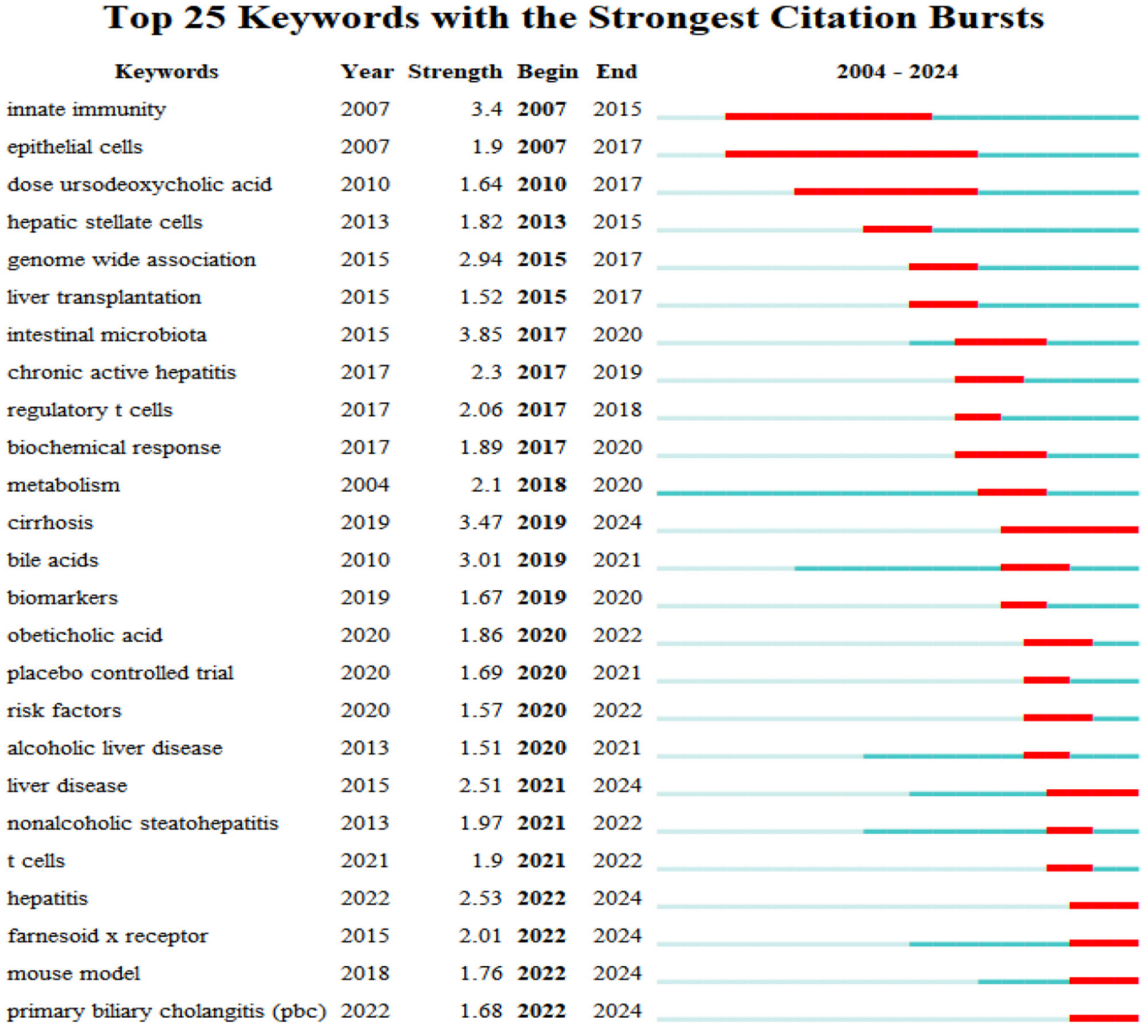
Top 25 most explosive keywords.

Keyword burst analysis is a practical tool to identify research hotspots (26). We performed a burst analysis of the top 25 keywords with the highest burst intensity in the literature using CiteSpace software (Figure 13). The top burst intensity over the 2017–2020 period corresponded to “intestinal microbiota” (3.85). During the same period, the keyword “biochemical response” was also accompanied by a burst (1.89), indicating that the focus of researchers during this period is on patients with PBC with a poor UCDA biochemical response, and that gut microbiota may become a potential treatment for such patients. This was followed by “cirrhosis” (3.47), “innate immunity” (3.4), “bile acids” (3.01), “genome wide association” (2.94), “hepatitis” (2.53), “liver disease” (2.51), “chronic active hepatitis” (2.3), “regulatory T-cells” (2.06), and “farnesoid X receptor” (2.01). “Epithelial cells” had the longest burst duration (2007–2017). The keywords that experienced explosive growth in the past 6 years were “cirrhosis,” “liver disease,” “hepatitis,” “farnesoid X receptor,” “mouse model,” and “primary biliary cholangitis,” indicating that the gut microbiota and liver diseases, including PBC, remain current and future research hotspots.

## Discussion

The concept of the “gut-liver axis” refers to the effects of bacteria on the development of liver disease through the enterohepatic circulation (29). Over the past two decades, considerable progress has been achieved, with research advancing from the experimental to the clinical stage by investigating the “gut-liver axis” and focusing on the role of the intestinal flora in the treatment of PBC (30). Due to the diverse types of gut microbiota and the influence of dynamic changes in the internal and external environment of the intestine, research on the gut microbiota in the field of PBC still faces challenges. Therefore, it is essential to understand the current situation and achieve further progress in this field. In this article, we extracted literature on the gut microbiota in the field of PBC from the Web of Science database and used the CiteSpace and VOSviewer software packages to sort and summarize the studies identified in this screening. We quantitatively analyzed these studies in terms of the publication quantity, country, author, institution, journal, and keywords and presented the results in a visual manner.

Our results showed that, over the past two decades, a total of 167 articles were published on intestinal flora and PBC, with the number of publications generally exhibiting an increasing trend year by year. This reflects the growing interest in intestinal flora as a potential target for PBC treatment compared to traditional drug therapy. Further research findings will emerge in this field in the future. Twenty-eight countries and regions contributed 167 papers on intestinal flora and PBC. China contributed the highest number of papers, although its influence and collaboration with other institutions needs to be strengthened. The United States and Italy followed as the next leading contributors. The research output of the United States was the greatest, with the highest total number of citations and total connectivity strength, demonstrating a strong influence in this field and close collaboration with other countries. Austria had the highest average number of citations. The institutional analysis showed that the University of California, Davis had the highest research output. Furthermore, two research institutions in China (Shanghai Jiaotong University and Capital Medical University) were among the top 10 research institutions in terms of the number of articles published, with the remaining institutions located in Western developed countries. This finding indicates that research institutions in China, Europe, and the United States hold leading positions within this research field. Close collaboration between research institutions in developed countries was verified, with the University of California, Davis at the center of the collaboration. While several Chinese research institutions demonstrated collaborative relationships, our results indicate a need to strengthen the collaboration. A significant correlation was observed between the size of the national and institutional contributions and that of individual authors.

Journal analysis revealed that the top 10 journals in terms of the publication volume were classified as either Q1 or Q2, with *Frontiers In Immunology* contributing the greatest number of articles, followed by *Liver International*, the *World Journal Of Gastroenterology*, and the *International Journal Of Molecular Sciences*. The most cited journal was the *Journal of Lipid Research*, followed by *Nature* and *Gut*. The top 10 cited journals were distributed in region 1, and the journal with the highest impact factor was *Nature* (IF: 50.5). The top 10 journals in terms of co-citations were also classified as Q1, with *Hepatology* and the *Journal of Hepatology* being frequently co-cited. This indicates that the results of studies on intestinal flora and PBC have received attention from high-quality journals, providing useful references for further research in this field and contributing to the advancement of this area of research. Literature published in molecular/biology/genetics journals is frequently cited in molecular, biology, and immunology journals, whereas those published in medical and clinical journals frequently cite literature from molecular biology, genetics, health, nursing, and other medical journals. This suggests a gradual shift from basic research toward the clinical application of research on gut flora and PBC.

Regarding the authorship analysis, Professor Gershwin and his team from the University of California, Davis (United States) made the most significant contribution in terms of publications and achieved the highest number of citations, highlighting their leadership in this field. The next greatest contributors were the teams led by Professor Ma Xiong and Professor Tang Ruqi from Shanghai Jiao Tong University (China). These author teams have close cooperation with each other, which reflects that strengthening cooperation can promote more scientific research output.

“Intestinal transport and metabolism of bile acids,” the most cited article, discusses the intestinal transport and metabolism of bile acids and, specifically, how intestinal flora influences the mechanism of action of bile acid signaling (18). “The gut microbial profile is altered in primary biliary cholangitis and partially restored after UDCA therapy” is the co-most cited article and is also the article with the highest outbreak intensity, which is indicative of its strong impact. The article demonstrated the impact of UDCA therapy by conducting a systematic comparative analysis of the gut microbiome in patients with PBC and in healthy controls, highlighting the gut microbiota as a potential therapeutic target and diagnostic marker for PBC (24). Based on the clustering of co-cited references, the time trend line chart (Figure 11) shows that the currently active clusters are #0 Microbiome, #3 Gut microbiome, and #7 Bile acids.

The co-occurrence analysis of high-frequency keywords highlights the frontiers and research hotspots in the related fields, providing valuable insights for identifying future research trends (11). Cluster analysis of keywords was performed to identify the frontiers and research hotspots in the study of intestinal flora and PBC. The top 10 keywords with the highest frequency were “primary biliary cholangitis,” “intestinal microbiota,” “primary sclerosing cholangitis,” “bile acids,” “ursodeoxycholic acid,” “cirrhosis,” “farnesoid X receptor,” “inflammatory bowel disease,” “risk factors,” and “liver disease.” These keywords were distributed in different clusters. “PBC,” “PSC,” “IBD,” and “risk factors” were distributed in the red cluster; this cluster mainly focuses on how intestinal flora participates in the pathogenesis of PBC.

PBC and PSC are a group of chronic immune liver diseases characterized by cholestasis (20). IBD is an immune-mediated chronic gastrointestinal disease comprising Crohn's disease (CD) and ulcerative colitis (UC). An abnormal immune response to the intestinal microbiota is considered the primary cause of IBD (31, 32). Patients with IBD exhibit a variety of extraintestinal manifestations, including liver function abnormalities, which are causally linked with the development of liver disease (33, 34). A two-sample Mendelian randomization analysis of genetic associations in genome-wide association studies revealed the following: IBD is a non-negligible risk factor for PBC and PSC; the risk of PSC is increased in UC and CD states; and CD increases the risk of developing PBC (35).

The gut-microbe-liver axis links the gut, gut microbes, the liver, and the gut mucosal barrier, which is composed of epithelial cells and serves as the gatekeeper for maintaining intestinal homeostasis (36). The surface of the epithelial cells is covered with a layer of mucus. The inner layer of mucus isolates the microorganisms from the epithelium, and the microorganisms are implanted in the outer layer of mucus, that is, they form the “mucosa-associated microbiota,” which acts directly on the intestinal barrier and thus has an impact on host immunity and metabolism (37). Immune cells assist epithelial cells in jointly guarding the intestinal mucosal barrier, with regulatory T-cells playing a key role in recognizing and protecting the gut from immune attacks by pathogenic microbes and harmful substances (38–40).

Regulatory T-cells also play a critical role in the pathogenesis of immune diseases such as IBD, PSC, and PBC (31, 41). In PBC, the complex immune response between the intestinal flora and the liver immune system disrupts the stable internal and external environment of the gut, resulting in an imbalance in the intestinal flora. The integrity of the intestinal mucosal barrier is breached, and permeability is increased. Dysregulated microorganisms and their metabolites are translocated through the gut-hepatic axis, and pathogenic microorganisms invade to produce lipopolysaccharide (LPS), which binds to toll-like receptors (TLRs) in the biliary epithelial cells, inducing an inflammatory cascade that leads to liver inflammation and fibrosis (7, 42, 43).

Bacterial infections are considered risk factors for the development of PBC. For example, *E. coli* infection can disrupt immune tolerance to mitochondrial autoantigens, producing AMAs, which are specific antibodies for PBC (44). These findings suggest the potential role of the intestinal flora as a therapeutic target to treat PBC. “Intestinal microbiota” and “liver disease” were two high-frequency keywords located in the green cluster, which focuses on the role of gut flora in the pathogenesis of other liver diseases. The intestinal-microbial-liver axis is the link between intestinal flora and the liver.

The intestinal flora play a role in PBC and other liver diseases, including autoimmune hepatitis, non-alcoholic fatty liver disease, non-alcoholic hepatitis, and hepatocellular carcinoma. Non-alcoholic liver disease is strongly associated with obesity, diabetes, and metabolic syndrome, and may progress to non-alcoholic hepatitis, cirrhosis, or hepatocellular carcinoma (45). A high-fat diet contributes to gut dysbiosis and interacts with the gut flora to trigger intestinal inflammation and insulin resistance (46, 47). Intestinal inflammation leads to dysfunction of the intestinal barrier and increased intestinal permeability, with bacterial or bacterial metabolites (e.g., endotoxins) translocating into the portal circulation and the liver. The subsequent induction of an inflammatory response through activation of TLRs leads to further progression of non-alcoholic fatty liver disease to non-alcoholic steatohepatitis (48).

Autoimmune hepatitis is an immune-mediated chronic liver disease which, along with PBC, is classified as an autoimmune liver disease and can progress to cirrhosis or hepatocellular carcinoma (49). Studies have shown that the pathogenesis of autoimmune hepatitis is associated with gut microbial dysbiosis (50, 51). Dysbiosis is involved in the development of autoimmune hepatitis through a variety of mechanisms, including the alteration of metabolite concentrations in the gut-liver axis, activation of various related signaling pathways, and modulation of immune responses in the gut and liver (52). Hepatocellular carcinoma is an advanced stage of various liver diseases. Dianne et al. (53) reported that intestinal flora and the metabolite LPS activated TLR4 to inhibit apoptosis of hepatocellular carcinoma cells and promote hepatocarcinogenesis in a mouse model of HCC.

“Bile acids,” “UDCA,” “farnesoid X receptor,” and other keywords were located in the blue cluster, which focuses on therapeutic targets for PBC. The main characteristic of cholestatic liver disease is excessive accumulation of bile acid (BA) (54), with high concentrations of BA inducing liver cell death and promoting liver fibrosis (55). The synthesis of BA is influenced by multiple factors, and farnesoid x receptor (FXR) is the most important receptor regulating BA homeostasis. FXR is highly expressed in bile duct and liver cells (56), regulating the enterohepatic circulation of BAs and preventing their toxic effects on liver and bile duct cells (57). Moreover, activation of the FXR pathway helps to increase the expression of tight junction proteins and antimicrobial peptides in intestinal epithelial cells, improve the integrity of the intestinal mucosal barrier, and alleviate liver fibrosis (58). Gut flora can regulate the synthesis of bile acids through the FXR signaling pathway (59), indicating the potential role of gut microbiota in the treatment of cholestatic liver disease. Gut flora, bile acids, and farnesoid X receptors are involved in the pathogenesis of many liver diseases, including cholestatic liver disease, non-alcoholic liver disease, and non-alcoholic steatohepatitis (48, 60, 61).

UCDA, originally obtained from bear bile, has a positive effect on PBC and various hepatobiliary diseases (62). As a hydrophilic secondary bile acid, UCDA reduces the toxicity of the bile acid pool by diluting the hydrophobicity of the secondary bile acids. It dissolves cholesterol-constituent gallstones and has anti-inflammatory, anticholestatic, antifibrotic, and antiapoptotic effects (63, 64). UCDA may alleviate PBC by altering the composition of the intestinal flora (25). These changes may be related to the direct effects of UDCA or may be secondary to improvement in cholestasis (65). Based on its pharmacologic profile, UCDA is also useful in the treatment of PSC (66), non-alcoholic steatohepatitis (67), cholestasis of pregnancy (68), and IBD (69). However, 40% of patients with PBC are unresponsive to UCDA (70). To address the issue of poor response to UCDA therapy, researchers developed obeticholic acid, an farnesoid X receptor agonist. In a double-blind randomized controlled trial of patients with PBC who had an inadequate response to UCDA, participants were treated with obeticholic acid (5–10 mg orally) for 12 months (71). The results showed decreased levels of alkaline phosphatase and total bilirubin compared to baseline, confirming the effectiveness of obeticholic acid in the treatment of PBC. However, as a first-generation farnesoid X receptor agonist, obeticholic acid exhibited poor bioavailability and adverse effects, such as dose-dependent pruritus (72). It should be pointed out that obeticholic acid can alleviate the imbalance of intestinal microbiota by activating FXRs (73). Therefore, it is anticipated that targeting the intestinal flora will represent a promising novel opportunity for the treatment of PBC.

The keyword “cirrhosis” was located in the yellow cluster, which focuses on cirrhosis. The inflammatory response is the primary mechanism by which the intestinal flora contribute to the pathogenesis of liver disease, a process triggered by the interaction between intestinal flora, the immune system, and the liver (74). Cirrhosis is an advanced manifestation of various types of liver diseases, including PBC, and studies in mouse models have shown that advanced liver fibrosis is associated with inflammation and increased microbial translocation (75). Gut microbes and metabolites translocate into the liver and bind to TLR, activating microbial LPS in Kupffer cells and hepatic stellate cells (43, 76). This leads to the production of collagen and other extracellular matrix components by hepatic stellate cells, thereby promoting liver fibrogenesis (77).

## Limitations

This study has some limitations. First, relative to other research fields, the research on intestinal flora and PBC is recent; therefore, the available literature is relatively limited. Second, during the data analysis, we merged some keywords with similar meanings, which may result in slight deviations but will not affect the overall results of this study. Thirdly, only literature published in English was extracted from the Web of Science database, without including literature published in other languages, which has certain limitations in comprehensively understanding the research on gut microbiota and PBC related fields. Finally, due to the time difference in the publication of relevant research literature, these documents cannot be cited in a timely manner. Overall, our study provides a reference for future research directions in the field of gut microbiota and PBC.

## Conclusion

This study applied Vosviewer and CiteSpace software to conduct the first bibliometric analysis on intestinal flora and PBC. We conducted a comprehensive analysis of 167 articles published in the past 20 years on gut microbiota and PBC, summarized the main countries, research institutions, and authors in this research field, identified the main published journals and literature, and found a correlation between gut microbiota and PBC through keyword analysis. The role of gut microbiota in the pathogenesis of PBC and the treatment of PBC will continue to be future research directions, and targets such as bile acids and farnesoid X receptors are also current research hotspots. The role of gut microbiota in the pathogenesis of PBC necessitates further exploration, which requires close cooperation between research institutions and scholars in different fields, such as molecular biology, immunology, genetics, clinical medicine, and pharmacy, to accelerate research and development in this field.

## Data Availability

The original contributions presented in the study are included in the article/supplementary material, further inquiries can be directed to the corresponding author.
